# Webinar report: stakeholder perspectives on informed consent for the use of genomic data by commercial entities

**DOI:** 10.1136/jme-2022-108650

**Published:** 2023-03-20

**Authors:** Baergen Schultz, Francis E Agamah, Cornelius Ewuoso, Ebony B Madden, Jennifer Troyer, Michelle Skelton, Erisa Mwaka

**Affiliations:** 1 National Human Genome Research Institute, National Institutes of Health, Bethesda, Maryland, USA; 2 Computational Biology Division, Department of Integrative Biomedical Sciences, Institute of Infectious Disease and Molecular Medicine, Faculty of Health Sciences, University of Cape Town, Cape Town, South Africa; 3 Medicine, University of the Witwatersrand, Johannesburg, Gauteng, South Africa; 4 Training, Diversity and Health Equity Office, Office of the Director, National Human Genome Research Institute, National Institutes of Health, Bethesda, Maryland, USA; 5 College of Health Sciences, Makerere University, Kampala, Uganda

**Keywords:** Ethics, Informed Consent, Ethics- Research

## Abstract

In July 2020, the H3Africa Ethics and Community Engagement (E&CE) Working Group organised a webinar with ethics committee members and biomedical researchers from various African institutions throughout the Continent to discuss the issue of whether and how biological samples for scientific research may be accessed by commercial entities when broad consents obtained for the samples are silent. 128 people including Research Ethics Committee members (10), H3Africa researchers (46) including members of the E&CE working group, biomedical researchers not associated with H3Africa (27), representatives from the National Institutes of Health (16) and 10 other participants attended the webinar and shared their views. Several major themes emerged during the webinar, with the topics of broad versus explicit informed consent, defining commercial use, legacy samples and benefit sharing prevailing in the discussion. This report describes the consensus concerns and recommendations raised during the meeting and will be informative for future research on ethical considerations for genomic research in the African research context.

## Background

Data sharing has many benefits, yet it also raises ethical issues. While some consent forms that discuss data sharing allow data to be used only for a specific study, others permit data to be shared and analysed more broadly. Broad consents are used to obtain an individual’s consent for the storage, maintenance and secondary research use of information or biospecimens. Guidance documents like the National Institutes of Health Data Sharing Policy, the European Union’s General Data Protection Regulations and the South African Protection of Personal Information Act (POPI ACT) tend to permit such broad consent. Furthermore, broad consent may be preferred and sometimes required by funding agencies because this maximises the utility of the investment. Secondary research can lead to future unforeseen scientific discoveries, including findings that lead to the improvement of healthcare; the enhancement of the capacity to predict disease risk, confirm a diagnosis and guide treatment; and/or the development of interventions that can indirectly benefit data contributors.^1^ Despite the potential benefits, there are concurrent ethical and practical challenges associated with data sharing. In particular, broad consent requires careful consideration with regard to ensuring that the participants are well informed about many different potential future uses of their data including who could or will have access. Researchers do not typically generate data from human samples to share for profit, rather, most samples are collected to answer a specific set of research questions. However, at the end of the research study, data generated from samples may be deposited in repositories. Depending on the terms of the consent, deposited data may be accessible to any researcher, including those associated with commercial entities.^2^ In some cases, data may even be available to the public. Concerns have been raised about sharing data with non-academic institutions, particularly if data are accessed in the future by commercial entities and may be used to create profitable drugs or interventions. This may be an especially sensitive issue for research done in disadvantaged, low-income, or marginalised communities. For instance, experiences with colonialism and the continuous plundering of resources in Africa by more powerful allies continue to colour relationships with commercial entities especially when they are from the Global North.^3^

Due to rapid scientific advances, researchers are often unable to predict the future use of samples at the time of sample collection when broad consent is obtained. For example, in the past, consent for research use was considered to include both not-for-profit and for-profit entities equally unless consent specified not-for-profit use (NPU) only.

Therefore, studies where samples were collected several years ago, or legacy studies, do not often contain a participant’s explicit preference regarding the use of their data by commercial (for-profit) entities. While following up primary analyses with unanticipated research can have scientific and public-health benefits and contribute to the collective good, some people argue consent is hardly informed if it is for future unknown use.^4^ If the advantages of data sharing are to be fully realised, further research and guidance are needed to address the important questions data sharing raises, including what principles ought to guide future use of data if participants’ expressed wishes for future use are not explicitly specified at the time of data collection.

The Human Heredity and Health in Africa (H3Africa) consortium was founded in 2011 as a result of discussions between the African Society of Human Genetics, the Wellcome Trust in the UK, and the US National Institutes of Health about the need to empower African genomic researchers.^5^ The purpose of H3Africa was to facilitate genomic research on the continent of Africa and provide appropriate infrastructure and ethical, social and legal frameworks around the same. The initiative consists of 51 studies that include population-based genomic studies across various diseases and disorders as well as research into the ethical, legal and social implications of genomics research on the African continent. As a condition of the awards, studies were required to obtain broad consent to share data generated from the studies for future research use. An H3Africa Data and Biospecimen Access Committee (DBAC) was established to assess requests for H3Africa data. Due to requests for use by commercial as well as academic institutions, the DBAC raised the question of what to do when consents were silent on this issue. While the H3Africa informed consent guidelines do recommend language regarding use by commercial entities, many H3Africa consents were obtained before those guidelines were written and do not explicitly address commercial use. In addition to this question, there were preceding concerns that some data from the African Variome Project may have been used without proper legal agreements with partner institutions, therefore, possibly violating consent. These data were used in the design of African-based genotyping arrays, including the H3Africa genotyping array. These products were designed and used by researchers, but ultimately, produced by and purchased from commercial entities.^6^ While these products were sold at cost and not for profit, it still raised significant concern and provoked a more than 2-year long debate within the H3Africa Consortium on the meaning, implications and impact of sharing samples and data with commercial entities, as well as additional consideration of benefit sharing and appropriate consent in genetics and genomic research.^7^

Because of this increased concern about the secondary use of genomic research data by commercial entities, H3Africa engaged ethics researchers to gain insight into the use of data for commercial purposes and develop an appropriate typology that can be used in the informed consent process.^7 8^ This activity involved the review of 26 consent documents from 18 H3Africa-funded projects and the findings revealed inconsistencies in the information disclosed to participants regarding the sharing of samples and data. Less than one-half of the projects had specific statements that samples would not be sold and that participants would not share in the profits from the development of therapeutic agents, and only 22% (4/18) used the term commercialisation. It is important to note that the views obtained in the H3Africa Report on Informed Consent and Commercialisation do not necessarily represent those of the wider communities in the countries involved.^7 8^

To further understand the issue, the H3Africa Ethics and Community Engagement (E&CE) Working Group organised a webinar for ethics committee members and biomedical researchers from various institutions throughout the African continent to discuss ethical challenges surrounding informed consent for the secondary use of data by commercial entities. This was not designed as a research study but as an information-gathering session. Because of the universality of the issues discussed, in this paper, we report and reflect on the opinions expressed during the webinar regarding whether and how samples should be accessed by commercial entities when broad consents that have been obtained are silent on this issue, and what other ethical considerations should be made.

### Main text

On 14 July 2020, the H3Africa E&CE working group hosted a 2-hour webinar that featured (1) an overview of the current genomics education resources that have been developed within H3Africa and (2) a discussion about the ethical challenges surrounding informed consent for the use of data by commercial entities.

**Figure 1:**
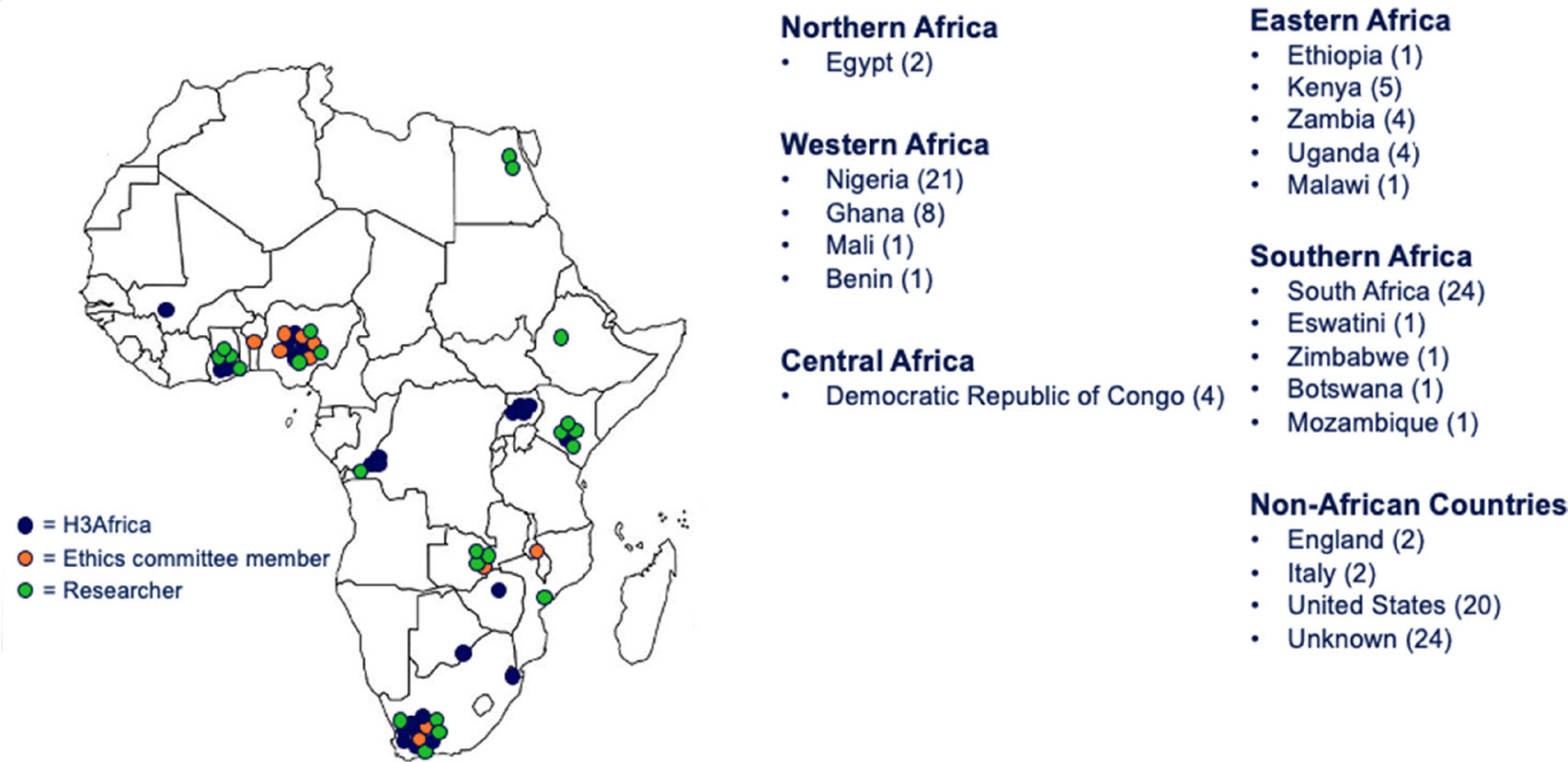
Sixteen African countries represented in the webinar.

The webinar was attended by 128 people including research ethics committee (REC) members (10), H3Africa researchers (46) including members of the E&CE working group, biomedical researchers not associated with H3Africa (27) and representatives from the National Institutes of Health (16). There were also 10 participants with unknown affiliations. There were 20 different countries represented at the webinar, including 16 African countries and 24 participants with an unknown country affiliation (figure 1). REC members were purposely selected and invited from RECs that were involved in the review of H3Africa research protocols and had attended previous consultation meetings hosted by the E&CE working group. Due to the open nature of the webinar, invitees were encouraged to share the invitation with their networks, which explains why the affiliation could not be captured for every participant of the webinar.

**Figure 2:**
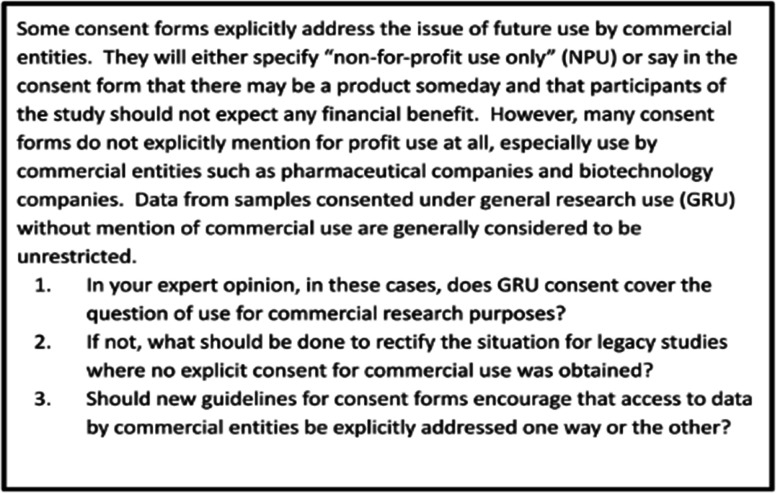
Guiding questions for webinar.

At the start of the webinar, several questions were shared to guide and focus the discussion (figure 2). All webinar participants were invited and encouraged to speak freely during the meeting or to share their thoughts in the chat feature. Attendees were informed and consented to both the chat and meeting being recorded to aid in summarising the major ideas from the webinar. Several major themes and recommendations emerged during the webinar, with the topics of broad versus explicit informed consent, defining commercial use, legacy samples and benefit sharing dominating the discussion. These are discussed in subsequent paragraphs. Importantly, rather than a systematic collection of opinions shared during the webinar, this report aims to encapsulate areas of consensus and majority opinion. Therefore, not every opinion expressed during the webinar is reflected in this report.

### Explicit consent

Some consent forms explicitly address the issue of future use by commercial entities. They will either specify ‘NPU only’ or indicate in the consent form that there may be a product in the future and that participants of the study should not expect any financial benefit. However, many consent forms do not explicitly mention for-profit use at all, especially use by commercial entities such as pharmaceutical and biotechnology companies. Historically, data from samples consented to under GRU without mention of commercial use are generally considered to be unrestricted. Webinar participants generally agreed that this standard is changing and that in the future data sharing with commercial entities should be an explicit component of the informed consent for use by commercial entities to be allowed. It was noted that this recommendation seems to be in line with local laws, such as South Africa’s POPI-Act.

### Defining ‘commercial use’

Meeting participants acknowledged that the concept of ‘commercial use’ is broad and has both negative and positive perceptions. As the discussion ensued it became apparent that the language used to describe ‘commercial use’ varies, as participants alternately spoke of ‘commercialisation’, ‘for profit use’ and ‘commercial use’. Participants agreed that the concept must be clearly explained in the informed consent documents, and it was suggested that past examples of commercial use should be included to help give a clear context. Furthermore, researchers should ensure that technical terms are clearly translated into local languages and simple enough that a layperson can understand the concept.

### Legacy samples

Much of the discussion focused on how to address ‘legacy samples’ where existing data were collected without participants’ explicit consent to data sharing with commercial entities. In cases where commercial use was not anticipated at the time of data gathering, it was generally agreed that, if possible, research participants should be recontacted for consent. However, webinar participants recognised that recontacting research participants would be costly and might not always be possible, and argued that in this scenario, researchers should seek guidance from the REC. It was suggested that the REC should deliberate on a case-by-case basis and consider the value and potential benefits to research participants and their communities when making decisions.

### Benefit sharing

It was clear during the meeting that there is a vast difference in opinions between the meeting participants when it comes to benefit sharing. While many participants recognised the potential advantages of sharing data for commercial purposes, one participant argued that the role of a researcher should be to heighten the benefits for humanity, which cannot be achieved through commercialisation. Some participants thought that benefits from commercial use should accrue to the people and communities who gave the samples. It is important to note that the benefits of genomic research may take several forms including, but not limited to monetary; technology transfer; research and infrastructural capacity building; reporting data genomic data back to the research participant; therapies derived from data that directly benefit the participants and/or community; community-based social projects and public education; providing feedback to research communities; and sustained access to funding. Explaining benefit sharing during informed consent was also discussed. Some webinar participants argued that as there is no guarantee that benefits will come back directly to original research participants, the informed consent should explicitly state that participants should not expect a direct benefit from their participation. However, many webinar participants supported including benefit sharing plans and communicating potential benefits to research participants. Many agreed that the question of how data sharing with commercial companies will benefit the communities of research participants needs to be considered. It was also suggested that fair benefit sharing should include discussions about fair access to any products. One participant argued that commercial entities need to explicitly commit to ensuring access to and affordability of products developed. Following these discussions, it is clear that the views of a variety of stakeholders that is, researchers, research regulators and communities on benefit sharing need to be further explored and understood.

## Discussions

In the last two decades, there has been an exponential increase in genomic research on the African continent, under the auspices of international collaborative initiatives such as the H3Africa Consortium, the African Genome Variation Project, the Neuropsychiatric Genetics of African Populations-Psychosis, and many more; as with most genomic studies, a lot of biological samples and genomic data have been collected.^5 9–11^ This rapid increase in the collection of genomic and other forms of digital big data is fast becoming one of the major ethical challenges to translational science in many of the international collaborative research studies due to the potential ethical issues around data access and use.

There is an increasing need to translate knowledge into products and policies, and this has created a range of policy challenges and other pertinent and legitimate concerns regarding genomic research including the role of REC, informed consent, community engagement, the end-users of the commercial products, potential harms and losses to the source population, public trust and expectations, benefit sharing and ownership, and intellectual property arrangements to mention a few.^7 8 12^ Whereas most stakeholders in the research enterprise feel that the movement of research outputs into the commercial realm is necessary for the benefit of humankind, many feel it is unethical to profit from a donor’s data and samples.^12–15^

Benefits of genomic research may take several forms including, but not limited to monetary; technology transfer; research and infrastructural capacity building; community-based social projects and public education; providing feedback to research communities; and sustained access to funding.^16–18^ Many stakeholders expect equitable and fair sharing with benefits trickling down to the community. However, there has been limited open debate on benefit sharing across the African continent, and the specific principles that should underlie benefit sharing relevant to African genomic research.^17^ One suggested principle is reciprocity since benefit sharing can reasonably be considered as one way of sharing research benefits with those who have contributed to its success.^19^ Further, studies show that there is limited awareness of the concept of benefit sharing by genomic researchers and research regulators in Africa and most countries in Africa lack comprehensive ethicolegal frameworks to guide benefit sharing.^13 20 21^ These challenges notwithstanding, stakeholders expect the benefit of sharing data in genomic research to be based on the principles of fairness, solidarity and reciprocity. H3Africa has tried to address some of these challenges by developing several guidance documents for genomic research.^7 8 22–26^

Based on these conversations and other engagements, the H3Africa Ethics Committee strongly recommends that for future studies, consent forms should explicitly address the secondary use of research data and/or samples by commercial entities. Ideally, if commercial entities are involved in the research from the outset, all material agreements should be adequately documented and related to participants. The nature of the commercial entities’ involvement, what data they will have access to, and how they will use such data should be clarified during the informed consent process.

In legacy situations where the original consent forms did not include a statement on sharing data with commercial entities, participants of the workshop proposed that the REC of record should decide on a case-by-case basis, rather than assuming that for-profit use would be acceptable. In light of the international move towards more open sharing of genomic data and the continued utility and use of legacy samples, further research is needed to assess participants’ understanding of the future use of samples and data with specific attention to expectations and preferences on which entities should have access to their data if they sign a consent form for broad data use.

## Conclusion

This report describes the opinions of REC members, H3Africa researchers, E&CE working group members, representatives from the National Institutes of Health, and other researchers from various African institutions who participated in a webinar organised by the H3Africa E&CE to discuss the issue of whether and how biological samples for scientific research may be accessed by commercial entities when broad consents obtained for the samples are missing. Some dominant themes that emerged during the discussion include explicit informed consent, defining commercial use, the REC weighing in on legacy samples and benefit sharing. For example, it was suggested that data sharing with commercial entities should be a core component of informed consent for genomic research going forward. This report should be of interest to African genomic researchers and informative for future research on ethical considerations for genomic research in the African research context. Studies are still required to assess participants’ preferences concerning whether and how commercial entities can use samples in the absence of (broad) consent.
